# Ethanol-Induced Dysbiosis and Systemic Impact: A Meta-Analytical Synthesis of Human and Animal Research

**DOI:** 10.3390/microorganisms13092000

**Published:** 2025-08-27

**Authors:** Luana Alexandrescu, Ionut Tiberiu Tofolean, Doina Ecaterina Tofolean, Alina Doina Nicoara, Andreea Nelson Twakor, Elena Rusu, Ionela Preotesoiu, Eugen Dumitru, Andrei Dumitru, Cristina Tocia, Alexandra Herlo, Daria Maria Alexandrescu, Ioana Popescu, Bogdan Cimpineanu

**Affiliations:** 1 Gastroenterology Department, “Sf. Apostol Andrei” Emergency County Hospital, 145 Tomis Blvd., 900591 Constanta, Romania; alexandrescu_l@yahoo.com (L.A.); eugen.dumitru@yahoo.com (E.D.); dr.andreidumitru@gmail.com (A.D.); cristina.tocia@yahoo.com (C.T.); ioanapop122@gmail.com (I.P.); 2 Medicine Faculty, “Ovidius” University of Constanta, 1 Universitatii Street, 900470 Constanta, Romania; tofoleandoina@yahoo.com (D.E.T.); ionelia.phb@yahoo.com (I.P.); 3 Pneumology Department, “Sf. Apostol Andrei” Emergency County Hospital, 145 Tomis Blvd., 900591 Constanta, Romania; 4 Internal Medicine Department, “Sf. Apostol Andrei” Emergency County Hospital, 145 Tomis Blvd., 900591 Constanta, Romania; alina.nicoara@365.univ-ovidius.ro (A.D.N.); andreea.purcaru@365.univ-ovidius.ro (A.N.T.); 5 Faculty of Medicine, Titu Maiorescu University, 040051 Bucharest, Romania; elenarusu98@yahoo.com (E.R.); alexandrescu_daria@yahoo.com (D.M.A.); 6 Department XIII, Discipline of Infectious Diseases, “Victor Babes” University of Medicine and Pharmacy Timisoara, 2 Eftimie Murgu Square, 300041 Timisoara, Romania; alexandra.mocanu@umft.ro; 7 Nephrology Department, “Sf. Apostol Andrei” Emergency County Hospital, 145 Tomis Blvd., 900591 Constanta, Romania; cimpineanub@yahoo.com

**Keywords:** ethanol, gut microbiota, dysbiosis, alcohol use disorder, gut–liver axis, microbiota–brain interaction, meta-analysis, rodent models, fecal microbiota transplantation, short-chain fatty acids

## Abstract

Background: Chronic ethanol consumption is a major global health concern traditionally associated with liver disease. Ethanol disrupts gut microbial communities, compromises intestinal barrier function, and contributes to hepatic, metabolic, and neurocognitive disorders. Methods: We conducted a systematic PubMed search and meta-analysis of 11 human and 19 animal studies evaluating ethanol-induced gut microbiota alterations. Studies were assessed for microbial diversity, taxonomic shifts, barrier integrity, and systemic effects. Effect sizes were calculated where possible, and interventional outcomes were examined. Results: Across species, ethanol exposure was consistently associated with reduced microbial diversity and depletion of beneficial commensals such as *Faecalibacterium*, *Lactobacillus*, *Akkermansia*, and *Bifidobacterium*, alongside an expansion of proinflammatory taxa (*Proteobacteria*, *Enterococcus*, *Veillonella*). Our analysis uniquely highlights discrepancies between human and animal studies, including opposite trends in specific genera (e.g., *Akkermansia* and *Bifidobacterium*) and the impact of confounders such as antibiotic exposure in human cohorts. We also demonstrate that microbiota-targeted interventions can partially restore diversity and improve clinical or behavioral outcomes. Conclusions: This meta-analysis highlights reproducible patterns of ethanol-induced gut dysbiosis across both human and animal studies.

## 1. Introduction

Alcohol consumption is deeply embedded in many cultures around the world. With more than 2.3 billion global consumers and approximately 75 million individuals suffering from alcohol use disorders (AUDs), ethanol (ethyl alcohol) poses a formidable burden on public health systems worldwide [1].

**Figure 1:**
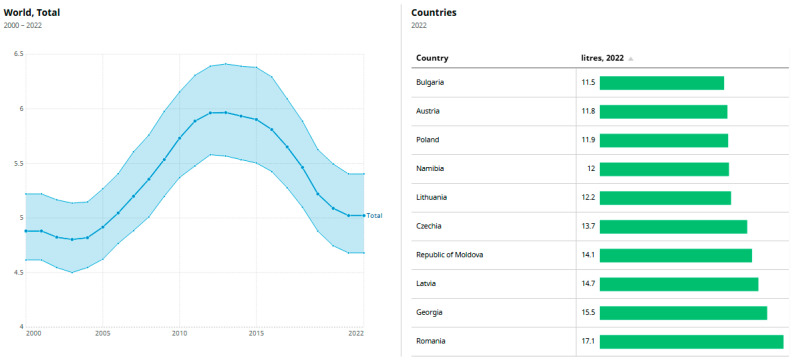
Total amount of alcohol consumed per adult (15+ years) over a calendar year, in liters of pure alcohol [1]. Data from WHO (Indicators).

Figure 1 displays the top nine countries ranked by total per capita alcohol consumption in 2022, based on WHO data. On the left, it shows the global average alcohol consumption (liters per capita) from 2000 to 2022, peaking around 2012 at nearly 6.5 L, followed by a gradual decline to about 5 L by 2022. The shaded area represents a confidence interval or range of variation across countries. On the right, a bar chart ranks countries by their 2022 per capita alcohol consumption, with Romania leading at 17.1 L, followed by Georgia (15.5), Latvia (14.7), and Moldova (14.1).

Although the liver is classically recognized as the primary target organ in alcohol-related morbidity, emerging research has accentuated the crucial role of the gastrointestinal (GI) tract, particularly the gut microbiome, in the initiation and progression of alcohol-related diseases [2].

### 1.1. Ethanol and Its Metabolic Impact on the GI Tract

**Figure 2:**
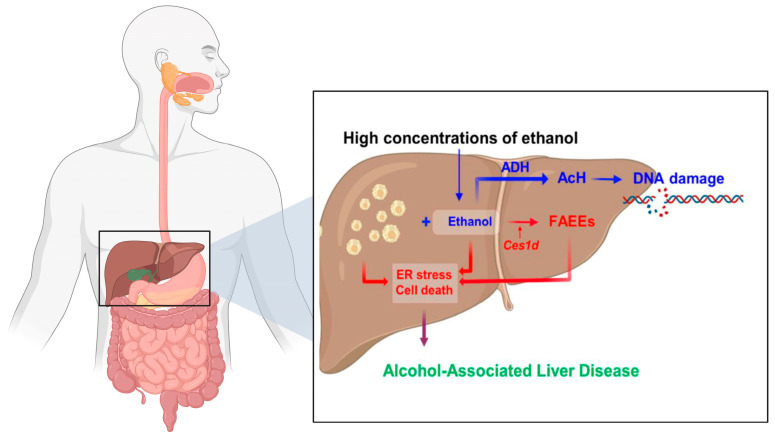
Oxidative and non-oxidative metabolism of ethanol. Data from Biorender [5].

Following oral ingestion, ethanol is rapidly absorbed in the stomach and proximal small intestine via simple diffusion and distributed throughout the body, including the distal GI tract, where its concentration mirrors that in systemic circulation [3]. Ethanol metabolism occurs via oxidative and non-oxidative pathways (Figure 2) [4].

**Figure 3:**
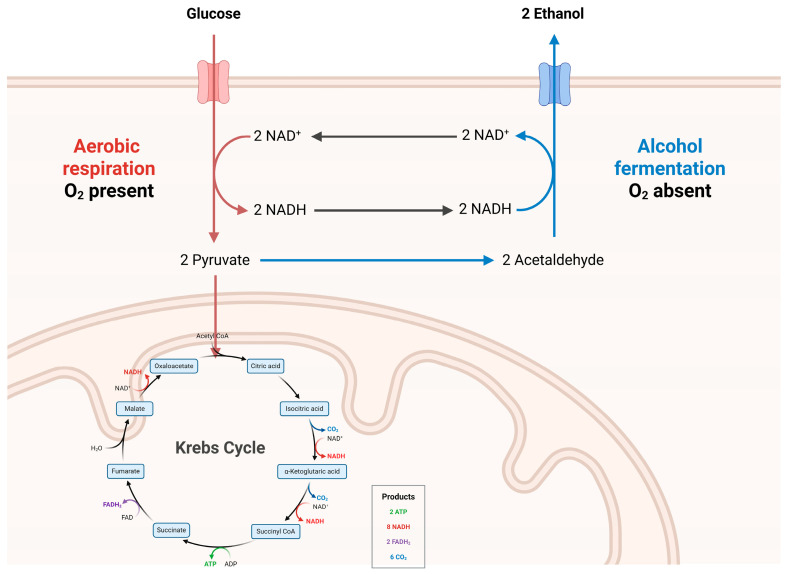
Ethanol and its metabolic impact. Created with Biorender [5]. Data from Madigan et al. [8].

Oxidative metabolism, mediated primarily by alcohol dehydrogenase (ADH) and cytochrome P450 2E1 (CYP2E1), yields acetaldehyde—a highly reactive and toxic intermediate implicated in tissue injury and carcinogenesis [6]. This is followed by conversion to acetate via aldehyde dehydrogenase (ALDH). While the liver carries out the majority of ethanol metabolism, significant enzymatic activity has also been observed in the epithelial cells of the small and large intestine [7]. The presence of metabolic machinery within the gut mucosa exposes the intestinal epithelium to locally generated acetaldehyde, particularly in the colon and rectum (Figure 3).

This localized metabolic activity is associated with the disruption of epithelial barrier function, or “leaky gut,” and has been shown to contribute to an increased risk of gastrointestinal cancers [7].

### 1.2. Alcohol-Induced Gut Barrier Dysfunction

One of the most deleterious effects of chronic ethanol consumption is the compromise of the gut epithelial barrier. Both ethanol and its primary metabolite, acetaldehyde, have been implicated in the disruption of tight junction proteins such as occludin and zonula occludens-1 (ZO-1), thereby increasing intestinal permeability [8]. Experimental studies in humans and rodents have demonstrated that both acute and chronic ethanol exposure leads to enhanced translocation of bacterial endotoxins such as lipopolysaccharides (LPSs) from the gut lumen into the portal circulation [9].

This breach of the epithelial barrier facilitates the entry of microbial-associated molecular patterns (MAMPs) into systemic circulation, which, in turn, activates hepatic Kupffer cells via toll-like receptors (e.g., TLR4), driving proinflammatory cascades that culminate in hepatic inflammation, fibrosis, and eventually alcoholic liver disease (ALD) [10].

### 1.3. Gut Microbiota Dysbiosis in Alcohol-Related Disease

**Figure 4:**
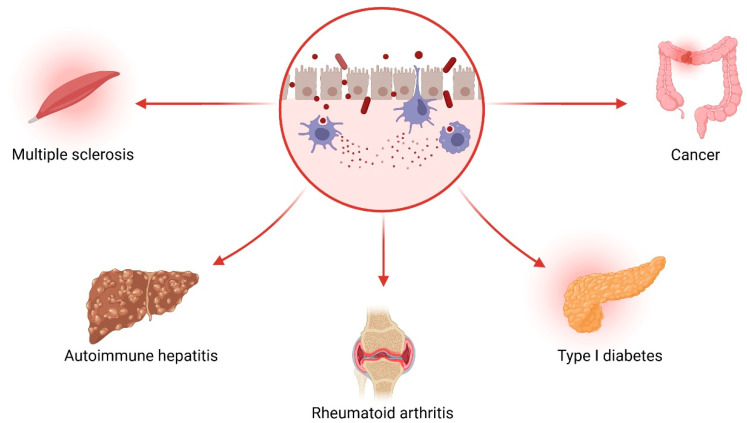
Microbial dysbiosis and its disease associations. Created with Biorender [5]. Data from Szychlinska et al. [13].

The gut microbiota, a complex ecosystem of trillions of microorganisms, plays an indispensable role in host metabolism, immune modulation, and barrier integrity [11]. This microbial imbalance has been implicated in the pathogenesis of several immune-mediated and metabolic diseases [12], including multiple sclerosis, autoimmune hepatitis, rheumatoid arthritis, type 1 diabetes, colorectal cancer, and other systemic disorders (Figure 4).

Chronic alcohol intake has been shown to significantly alter the composition, diversity, and function of the gut microbiome, a phenomenon broadly described as dysbiosis [10]. Both preclinical and clinical models have demonstrated ethanol-induced microbial shifts characterized by a decline in beneficial commensals, such as *Faecalibacterium prausnitzii*, *Lactobacillus*, and *Bifidobacterium*, accompanied by an overgrowth of potentially pathogenic genera such as *Proteobacteria*, *Clostridium*, and *Fusobacterium* [14,15].

Preclinical studies using ethanol-fed rodent models have revealed marked increases in bacterial overgrowth in the upper small intestine and significant alterations in the cecal microbiota, including elevated levels of *Bacteroides* and *Verrucomicrobia* alongside reduced *Firmicutes* [16]. These microbial shifts have been associated with suppressed expression of host antimicrobial peptides such as Reg3β and Reg3γ, exacerbating mucosal vulnerability and bacterial translocation [17]. In humans, ethanol consumption has similarly been linked to decreased fecal concentrations of butyrate-producing bacteria and short-chain fatty acids (SCFAs), which are critical for maintaining intestinal homeostasis and mucosal healing [18].

### 1.4. The Gut–Liver–Brain Axis: A Triangular Pathophysiological Circuit

**Figure 5:**
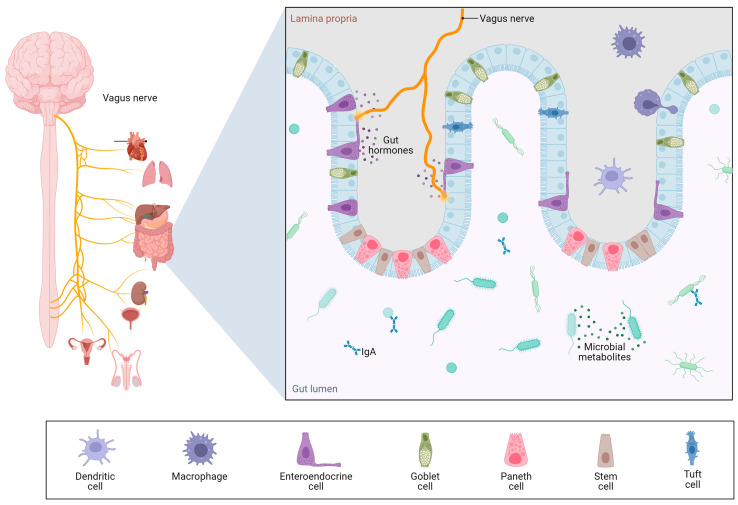
Gut–brain axis. Created with Biorender [5]. Data from Gonzalez-Santana et al. [21].

Beyond its hepatic consequences, ethanol-induced dysbiosis and gut barrier dysfunction appear to play central roles in the systemic complications of alcohol use disorder [19]. The gut–liver axis is now expanded to include the brain, forming a complex gut–liver–brain axis [20], in which microbial dysbiosis, intestinal permeability, and inflammatory signaling converge to influence neuropsychiatric outcomes (Figure 5).

Evidence indicates that microbial-derived products crossing a compromised gut barrier not only exacerbate liver injury but also reach the central nervous system (CNS), where they may trigger neuroinflammation and contribute to cognitive impairments observed in AUD [22]. Furthermore, ethanol-induced reductions in microbial synthesis of vitamins such as thiamine can lead to Wernicke–Korsakoff syndrome and other neurodegenerative manifestations [23].

### 1.5. Therapeutic Potential of Targeting the Gut Microbiota

Given the intimate interplay between ethanol metabolism, gut microbiota dysbiosis, and systemic inflammation, restoring microbial homeostasis has emerged as a promising therapeutic avenue. Several studies have demonstrated that administration of probiotics or fecal microbiota transplantation (FMT) from healthy donors can ameliorate ethanol-induced gut dysbiosis and its sequelae. In murine models, treatment with *Lactobacillus rhamnosus* or dietary oats improved microbial balance and intestinal barrier integrity, while in humans, probiotic supplementation increased beneficial bacterial counts and improved liver enzyme profiles [24].

These interventions hold promise not only for mitigating the progression of ALD but also for alleviating neurocognitive symptoms in patients with AUD [25]. The metabolomic profile of the gut microbiota, including SCFAs and bile acids, may also serve as biomarkers for disease severity and response to therapy [26].

Thus, ethanol-induced disruptions in gut microbiota composition and function constitute a key mechanism linking alcohol consumption to systemic diseases, particularly liver injury and neurocognitive disorders. Through alterations in microbial taxa, metabolite profiles, and intestinal permeability, ethanol sets the stage for a cascade of inflammatory and fibrotic events with implications that extend far beyond the liver. This meta-analysis seeks to consolidate current evidence on the bidirectional relationship between ethanol intake and gut microbiota dysbiosis with the aim of elucidating mechanistic pathways and evaluating the therapeutic potential of microbiota-targeted interventions [27].

Unlike prior reviews on ethanol-induced dysbiosis, our study integrates data from both human and animal studies to generate meta-aggregated effect sizes and highlight translational patterns. Additionally, we provide a direct comparative analysis between human and animal models and evaluate the impact of microbiota-targeted interventions, offering insights into mechanistic pathways and therapeutic potential that were not addressed in previous reviews.

## 2. Materials and Methods

### 2.1. Strategy for Searching the Literature

A systematic search was conducted using the PubMed database to identify original research articles evaluating the effects of ethanol exposure on gut microbiota in both human and animal studies. The search included combinations of MeSH terms and free-text keywords such as “ethanol”, “alcohol”, “gut microbiota”, “intestinal microbiome”, “dysbiosis”, “humans”, “mice”, “rats”, and “rodent models” [28]. Boolean operators “AND” and “OR” were applied to optimize the yield. Searches were limited to articles published in English [29]. No restrictions were placed on publication date.

**Figure 6:**
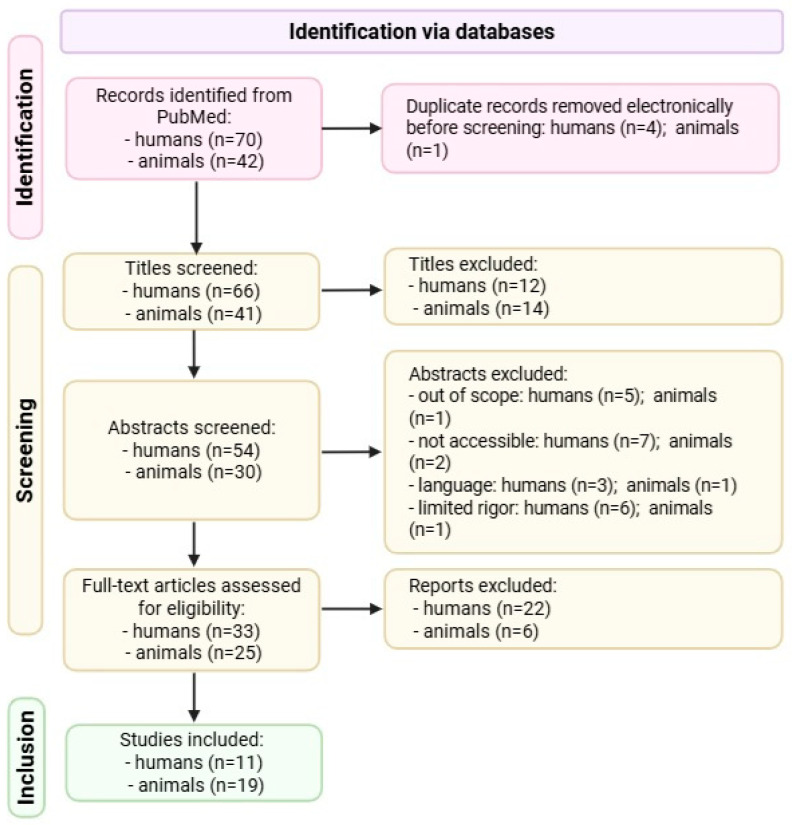
PRISMA flow diagram. Created with Biorender [5]. Data from www.prisma-statement.org, accessed on 12 June 2025 [30].

The full selection process is detailed in the PRISMA flow diagram (Figure 6) [30].

The search initially identified a total of 112 records, including 70 human studies and 42 animal studies. After the removal of duplicates (4 human, 1 animal), 107 records were screened by title. This was followed by abstract screening (humans: 54; animals: 30) and subsequent full-text review (humans: 33; animals: 25) to determine final eligibility. Based on predefined inclusion and exclusion criteria, 11 human studies and 19 animal studies were included in the final analysis.

### 2.2. Inclusion and Exclusion Criteria

Inclusion criteria were defined separately for human and animal studies:

- Human studies: eligible if they investigated individuals with chronic alcohol use or alcohol dependence, assessed gut microbiota composition using sequencing or molecular tools, and included a comparator group (e.g., healthy controls or non-drinkers).
- Animal studies: included if the animals were exposed to ethanol via drinking water, liquid diet, vapor exposure, or gavage and reported microbiota outcomes assessed via validated methods (e.g., 16S rRNA sequencing, qPCR).
- There were no geographical restrictions in study selection.

Exclusion criteria applied to both domains:

- Lack of a control group, absence of microbiota outcome data;
- Non-ethanol-related interventions (e.g., antibiotics alone, prebiotics);
- Language other than English;
- Limited methodological rigor;
- Inaccessible full text.

Specifically, 12 human titles and 14 animal titles were excluded during the title screening phase. Abstract-level exclusions included scope mismatches, inaccessible papers, language barriers, and insufficient study design. At the full-text level, 22 human and 6 animal studies were excluded primarily due to insufficient control, unclear ethanol effect attribution, or poor methodological quality.

Ethanol was selected as the focus of this analysis because it represents the predominant form of alcohol consumed globally and is the principal agent responsible for alcohol-related disease. Moreover, ethanol-based rodent models are well characterized and have been extensively validated to reproduce key features of alcohol-induced dysbiosis.

### 2.3. Screening and Data Extraction

All screening steps—titles, abstracts, and full texts—were performed independently by two reviewers, with disagreements resolved by discussion. Data extracted included population characteristics (species, strain, age, sex), ethanol exposure parameters (dose, route, and duration), microbiota analysis method (e.g., sequencing platform, region targeted), and key outcomes (diversity metrics, taxonomic shifts, functional alterations).

For human studies, additional variables, such as alcohol consumption history, diagnostic criteria, sample source (e.g., feces, mucosal biopsy), and reported comorbidities, were recorded.

The number of included studies was limited by the challenge of identifying randomized controlled trials and high-quality observational studies with sufficient methodological rigor and heterogeneity to allow for meaningful synthesis. Many studies were excluded due to the absence of control groups, inadequate microbiota outcome data, or failure to meet minimum quality criteria. The final 11 human and 19 animal studies therefore represent the best available evidence suitable for robust comparative analysis.

### 2.4. Quality Assessment

Animal studies were assessed using the SYRCLE risk of bias tool [31]. Most studies reported randomization, ethical compliance, and sample size estimates but often lacked details on blinding and environmental controls.

Human studies were evaluated using the Newcastle–Ottawa scale, focusing on study selection, comparability, and outcome assessment [32]. While most human studies adequately defined exposure and outcome, variability was noted in controlling for confounders such as diet and medication use.

### 2.5. Data Synthesis and Analysis

Given the diversity in experimental designs and microbiota outcome reporting, both narrative synthesis and quantitative comparison were employed. Microbial diversity (alpha and beta), shifts in major phyla (e.g., Firmicutes, Bacteroidetes, Proteobacteria), and functional signatures (e.g., SCFA production, bile acid metabolism) were synthesized descriptively.

Where applicable, summary measures such as mean differences and confidence intervals were calculated for comparative analysis between ethanol-exposed and control groups. Results from both human and animal studies were tabulated and analyzed separately to preserve biological relevance.

No funding was utilized in the design, conduct, analysis, or preparation of this article. All research activities were carried out independently by the authors without financial support from public or private institutions.

## 3. Results

Table 1 and Table 2 below present the core studies included in this meta-analysis, offering a structured overview of ethanol-induced gut microbiota alterations in both humans and animal models. Table 1 summarizes 11 human studies, highlighting participant characteristics, diagnostic categories, intervention types, and key microbiota outcomes. Table 2 compiles findings from 19 animal studies involving rodent models, detailing ethanol exposure protocols, microbial shifts, and associated physiological or behavioral effects.

### 3.1. Human Cohort Findings: Microbiota Diversity and Clinical Outcomes

To assess the impact of ethanol exposure on gut microbiota in humans, we included 11 eligible studies that met all inclusion criteria. These studies spanned various designs, including randomized controlled trials, observational studies, and interventional protocols involving fecal microbiota transplantation, probiotics, abstinence programs, or pharmaceutical agents.

#### 3.1.1. Population and Study Characteristics

**Figure 7:**
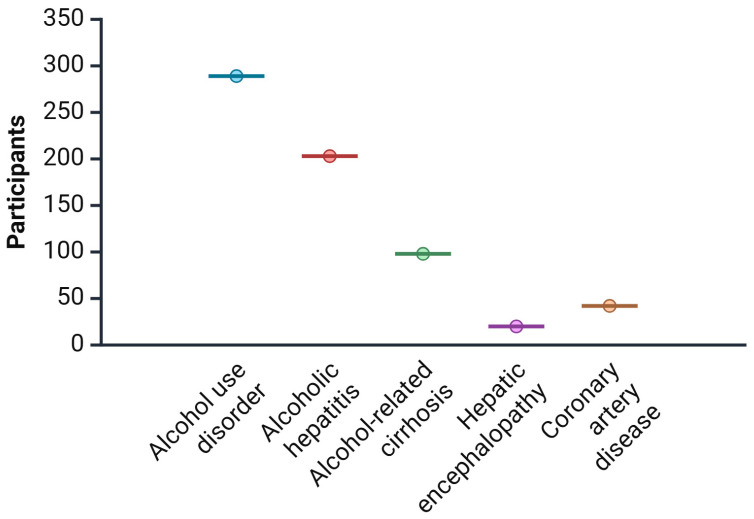
Breakdown of participant diagnoses across the 11 human studies.

The selected studies involved a total of 702 participants aged between 18 to 65 years, with most subjects being male (Table 3). Participants included individuals diagnosed with AUD, ARC, AH, and related comorbidities. Control groups varied across studies, comprising healthy individuals, placebo groups, or standard-of-care cohorts (Figure 7).

#### 3.1.2. Effect Sizes for Human Studies

Table 4 summarizes the calculated effect size estimates for the included human studies.

Among the studies with sufficient data, Du et al. [33] demonstrated large negative effect sizes for cognitive outcomes (MoCA: *d* = −0.98; MMSE: *d* = −1.25), indicating worse performance in the AUD group compared to healthy controls. Dedon et al. [34] showed a small-to-moderate effect (*d* = 0.39) in favor of placebo for percent drinking reduction, while Bajaj et al. [36] reported a small effect (*d* = 0.29) favoring FMT over placebo for alcohol craving (ACQ-SF). Philips et al. [38] revealed a large effect (*d* = 0.69) for improved 90-day survival with FMT compared to high-dose probiotic infusion. Similarly, Zhang et al. [40] reported moderate-to-large effects for reductions in liver enzymes (AST: *d* = 0.66; γ-GT: *d* = 0.87) with BC99 treatment compared to placebo. Han et al. [43] observed a small effect size for serum LPS reduction with probiotics (*d* = 0.18). The remaining studies (Zhang et al. [35], Amadieu et al. [37], Muthiah et al. [39], Lang et al. [41], and Haas et al. [42]) did not provide sufficient outcome data to compute effect sizes.

In most cases, beneficial shifts, such as increased abundance of *Lactobacillus* or decreased *Enterobacteriaceae*, were observed during or immediately after the intervention period, with limited follow-up extending beyond 4–8 weeks (Table 5).

As such, the long-term persistence of these effects remains uncertain. Notably, Wang et al. [62] provided limited post-treatment data suggesting partial reversion of microbial profiles, implying that colonization by introduced or promoted taxa may be transient in the absence of sustained intervention.

#### 3.1.3. Microbiota Alterations

**Figure 8:**
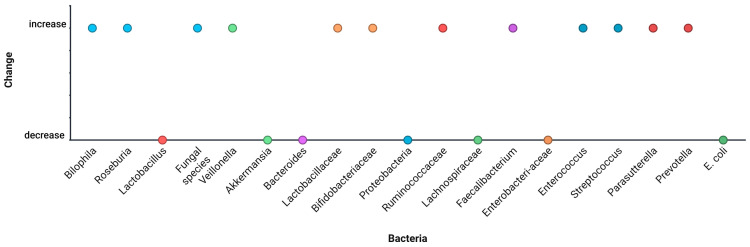
Meta-aggregated taxonomic alterations across all human studies. Data from [33,34,35,36,37,38,39,40,41,42,43].

Human studies reported a consistent pattern of dysbiosis following ethanol exposure (Table 6):

- Decreases: Ruminococcaceae, Lachnospiraceae, Faecalibacterium, Akkermansia, Bifidobacterium;
- Increases: Enterococcus, Streptococcus, Veillonella, Proteobacteria (especially Enterobacteriaceae) (Figure 8).

Changes in microbial diversity (α-diversity and β-diversity) were often correlated with clinical severity, systemic inflammation (↑ IL-6, TNF-α, LBP), and psychological symptoms (↑ craving, ↓ cognition).

### 3.2. Animal Model Findings

The studies analyzed employed a diverse array of animal models, with mice being the predominant species (n = 13), particularly the C57BL/6J, BALB/c, and ICR strains. Rats were used in five studies, most commonly the Wistar and Sprague Dawley strains. One study uniquely utilized human fecal microbiota transferred into rats, bridging preclinical and translational paradigms.

**Figure 9:**
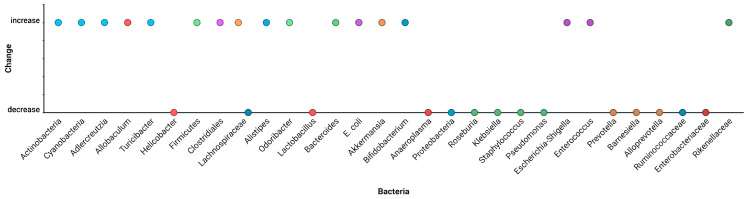
Meta-aggregated taxonomic alterations across all animal studies. Data from [45,46,47,48,49,50,51,52,53,54,55,56,57,58,59,60,61,62].

Ethanol administration routes varied, with the Lieber–DeCarli liquid diet being the most commonly employed method for chronic exposure. Other models included drinking water supplementation (gradually increasing or fixed concentrations), oral gavage, vapor exposure, intermittent binge-like exposure, and ethanol combined with a high-fat diet (HFD). The ethanol doses ranged from 5% to 56%, with exposure durations from 4 weeks to 12 weeks, or longer for chronic use. One study mimicked acute exposure using a binge protocol, while another included subacute vapor exposure to simulate inhalation effects (Table 7, Figure 9).

A consistent observation across experimental models was the reduction in beneficial commensal bacteria. Notably, *Lactobacillus* species were significantly depleted in at least six studies, suggesting a reproducible pattern of sensitivity to ethanol’s disruptive effects. Other commensals, such as *Akkermansia* and *Allobaculum*, were frequently altered, with some interventions promoting their abundance. These bacteria are known for their roles in maintaining mucosal integrity, modulating immune responses, and supporting metabolic homeostasis. Additionally, short-chain fatty-acid-producing bacteria, including *Butyricimonas* and members of the *Ruminococcaceae* family, were found to decline in abundance. The loss of these SCFA producers likely contributes to impaired gut barrier function and heightened susceptibility to inflammation.

Conversely, ethanol exposure favored the overgrowth of potentially pathogenic or proinflammatory taxa. An increase in the phylum *Proteobacteria*, particularly *Enterobacteriaceae* family members, was a prominent finding, especially in the studies by Hendrikx et al. [53] and Chen et al. [54]. Similarly, other opportunistic bacteria, such as *Helicobacter*, *Fusobacterium,* and *Escherichia coli*, exhibited increased relative abundance under ethanol influence. Several studies also reported an elevated *Firmicutes*-to-*Bacteroidetes* ratio, most notably in the work by Daaz-Ubilla et al. [44].

Only Wang et al. [47] investigated fungal components, identifying ↑ *Saccharomyces* and *Kurtzmaniella*, ↓ *Candida,* and a significantly reduced fungal-to-bacterial ratio.

In parallel, ethanol-induced dysbiosis was frequently associated with compromised intestinal barrier integrity. Elevated levels of biomarkers like intestinal fatty acid binding protein (i-FABP) and LPS were observed, suggesting increased gut permeability and endotoxin translocation into systemic circulation. This barrier dysfunction likely contributes to the systemic inflammation and hepatic damage documented in multiple studies, including those by Daaz-Ubilla [44], Hendrikx [53], and Wang [47].

Table 8 highlights notable discrepancies in microbial responses to ethanol exposure between human and animal studies.

*Akkermansia* decreased in humans [34] but increased in several animal studies [47,56,57,59,61]. In his research, Yan et al. [63] mentions that this might be due to species-specific microbiome dynamics or the absence of confounding clinical factors in animal models. Similarly, *Bacteroides* and *E. coli* decreased in human studies [34,42,43] but increased in rodents [48,62]. Nguyen et al. [64] concluded that this might be because of various confounding factors ranging from diet to exposure to pathogens. Contrasting trends were also seen for *Lachnospiraceae*, *Prevotella*, and *Roseburia*.

#### 3.2.1. Effect Sizes for Animal Model Studies

Table 9 presents the calculated effect size estimates for the 19 animal studies included in this analysis.

Several studies demonstrated very large effect sizes, particularly those measuring ethanol intake, liver injury markers, and behavioral outcomes. For instance, Díaz-Ubilla et al. [44], Wang et al. [47], Xia et al. [51], and Thoen et al. [58] reported Cohen’s *d* values > 4 for key outcomes such as ethanol intake and serum ALT. Behavioral assessments, including the open-field test, elevated plus maze, forced swim test, and tail suspension test, also consistently showed large effects (e.g., *d* > 1) in studies such as those by Xiao et al. [46], Xu et al. [45], and Wang et al. [62], indicating pronounced anxiety- and depression-like behaviors associated with alcohol exposure or fecal microbiota transplantation from alcohol-dependent subjects. Biochemical markers also revealed large differences across groups (Han et al. [55], Li et al. [48], Cunningham et al. [61]).

#### 3.2.2. Intervention Outcomes

**Figure 10:**
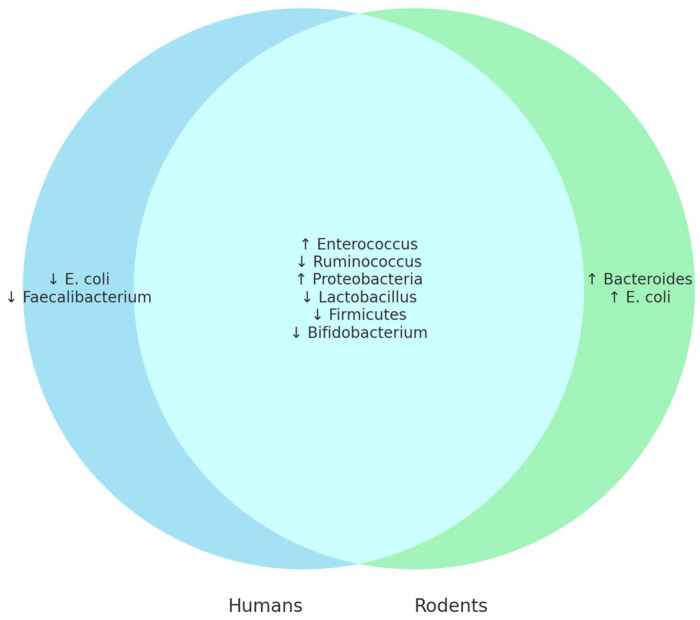
Venn diagram showing differences and similarities in the change in intestinal microbiota between humans and animals. ↑ indicates an increase in bacterial abundance or clinical parameter; ↓ indicates a decrease.

Several studies evaluated interventions aimed at reversing or mitigating alcohol-induced dysbiosis (Figure 10).

Probiotic therapy: *Lactobacillus casei* supplementation reversed microbial shifts (↑ *Lactobacillus*, ↓ *E. coli*) and corrected iron metabolism disturbances by reducing ferritin and hepcidin and restoring transport proteins (DMT1, FPN1) [48]. In the study by Jiang et al. [59], co-treatment with probiotics partially restored the gut microbiota and improved barrier function.

Studies incorporating ethanol withdrawal showed partial or complete recovery of microbiota profiles. For example, Xia et al. [51] documented reversibility of SCFA-producing bacteria, while Yang Fan et al. [52] demonstrated colonic restoration of beneficial taxa and functional metabolic pathways after ethanol cessation.

FXR-deficient mice [53] exhibited worsened liver pathology and microbial imbalance, pointing to nuclear receptor pathways as therapeutic targets.

## 4. Discussion

This meta-analysis consolidates evidence from 11 human and 19 animal studies to highlight the consistent, ethanol-induced perturbations in gut microbiota composition, diversity, and function. Notably, both categories of studies demonstrated convergence on hallmark microbial changes, suggesting robust and translationally relevant biological patterns.

Multiple human studies (Table 1) have demonstrated consistent taxonomic alterations associated with ethanol exposure. Specifically, a significant reduction in *Lactobacillus* abundance was reported by Du et al. [33], Philips et al. [38], and Bajaj et al. [36]. Comparable findings were also observed in animal models, as evidenced by the results of Li et al. [48] and Yang et al. [55] (Table 2). These reductions are significant given *Lactobacillus*’s role in maintaining mucosal integrity and immune regulation. Consistent with our findings, Chancharoenthana et al. [65] reported that chronic alcohol exposure in mice suppressed *Lactobacillus* abundance and impaired tight junction expression, leading to increased gut permeability and endotoxemia.

Similarly, *Akkermansia*, a mucin-degrading genus linked to metabolic and gut barrier health, declined in both Dedon et al. [34] and Jiang et al. [59], while probiotic or dietary interventions restored its abundance. These trends align with observations by Wei et al. [66], who found that *Akkermansia* supplementation attenuated ethanol-induced steatosis and inflammation in murine models.

Microbial diversity also emerged as a central theme. Decreased alpha-diversity was reported in severe AUD cohorts [37,41], echoing findings in several animal models where chronic ethanol exposure reduced microbial richness [47,60]. Parallel work by Capurso et al. [67] supports these outcomes, showing that long-term alcohol use leads to ecological instability in the microbiome and favors pathogenic overgrowth.

A notable distinction between human and animal studies lies in the complexity of comorbid conditions. While animal models often isolate ethanol as the primary variable, human studies [36,38] contend with layered pathologies such as hepatic encephalopathy, cirrhosis, or psychiatric symptoms. Despite these confounders, both domains recorded elevated *Proteobacteria*, especially *Enterobacteriaceae*, a marker of dysbiosis and inflammation. This is consistent with findings by Smirnova et al. [68], who demonstrated increased *Proteobacteria* in alcoholic hepatitis patients and linked it to systemic inflammation.

A notable divergence was noted in the abundance of *Escherichia coli* between human and animal studies. In humans, a reduction in fecal *E. coli* counts has been consistently observed, particularly in hospitalized patients with alcoholic hepatitis, which may be attributed to prior exposure to antibiotics, altered bile acid profiles, or impaired mucosal immunity secondary to liver dysfunction. These factors can selectively suppress facultative anaerobes such as *E. coli* within the gut lumen. In contrast, rodent models exhibited an increase in *E. coli* abundance following chronic ethanol exposure. This discrepancy may be attributed to species-specific differences in intestinal physiology and microbiota resilience, as well as the more controlled experimental conditions in animal studies. In rodents, ethanol-induced disruption of tight junctions and increased intestinal permeability may facilitate colonization and expansion of *E. coli* and other opportunistic taxa, which thrive in the inflamed and oxygen-enriched microenvironment of a compromised gut barrier.

Interventional outcomes offer additional insights. In humans, FMT significantly improved microbial diversity and clinical endpoints [36,38], while in animals, similar benefits were seen with *Lactobacillus casei* or *Clostridium butyricum* supplementation [48,49]. Comparable benefits have been documented by Pu et al. [69], who reported that *Clostridium butyricum* ameliorated ethanol-induced gut and liver injury via TLR4/NF-κB pathway inhibition.

Another important factor contributing to variability in the findings is the heterogeneity in microbiota sequencing techniques and analysis pipelines across the included studies. While the majority of both human and animal studies utilized 16S rRNA gene sequencing, there was significant inconsistency in the specific hypervariable regions targeted (V3–V4, V4 alone, or V1–V3). Moreover, differences in DNA extraction methods, sequencing platforms, and downstream bioinformatics pipelines (OTU vs. ASV-based clustering, use of different reference databases) further complicate direct cross-study comparisons. A small number of studies employed shotgun metagenomic sequencing, offering higher resolution but introducing additional variability in functional and compositional analyses.

While several studies in this review reported neurobehavioral alterations associated with dysbiosis, it is important to note that most of these findings remain correlative. Direct evidence linking specific microbial metabolites to neurobehavioral outcomes is still limited. A few preclinical studies have demonstrated potential causality; for instance, SCFA supplementation has been shown to restore GABAergic signaling and ameliorate anxiety-like behavior in germ-free or antibiotic-treated rodents. However, such mechanistic insights have not yet been conclusively validated in human cohorts.

Observed behavioral outcomes associated with microbial alterations support the translational relevance of findings between preclinical and clinical studies. Rodents exposed to dysbiotic microbiota displayed depressive and anxiety-like behaviors [45,46], paralleling observations of increased craving and reduced cognition in AUD patients [34,37]. These findings are echoed by Nikel et al. [70], who identified correlations between gut dysbiosis and anxiety scores in AUD cohorts.

## 5. Limitations

One important limitation of this meta-analysis lies in the heterogeneity of control groups across included studies. Control cohorts ranged from healthy individuals with no alcohol exposure to placebo-treated or standard-of-care populations, introducing variability in baseline microbiota composition and systemic parameters. While this diversity does not invalidate the findings, it represents a potential source of bias that must be acknowledged in the interpretation of results. In addition, significant heterogeneity was observed in other aspects of study design, including ethanol dosing regimens, intervention durations, and microbiota analysis techniques. Specifically, studies employed differing routes and concentrations of ethanol administration (e.g., oral gavage vs. voluntary intake), and microbiota characterization ranged from 16S rRNA gene sequencing (with different hypervariable regions) to shotgun metagenomics.

## 6. Conclusions

This meta-analysis provides compelling evidence that ethanol exposure induces consistent and functionally significant alterations in the gut microbiota across both human and animal studies. Despite differences in methodology, host physiology, and experimental design, the findings converge on key microbial signatures: a depletion of beneficial commensals such as *Faecalibacterium*, *Akkermansia*, *Lactobacillus*, and *Bifidobacterium*, alongside an expansion of proinflammatory taxa like *Proteobacteria*, *Enterococcus*, *Veillonella*, and *Escherichia-Shigella*. These microbial alterations are accompanied by reduced diversity and a depletion of short-chain fatty-acid-producing organisms, which collectively contribute to compromised gut barrier integrity, increased intestinal permeability, and systemic inflammation.

In humans, dysbiosis was associated with alcohol use disorder, alcoholic hepatitis, cirrhosis, and neurocognitive symptoms such as increased craving and cognitive impairment. In animal models, microbial shifts were not only reproducible but also shown to mediate behavioral changes, including anxiety and depressive-like states.

Interventional studies further reinforce the modifiability of the gut microbiome. Probiotic supplementation, fecal microbiota transplantation, and ethanol abstinence were shown to restore microbial diversity, rebalance key taxa, and improve clinical or behavioral outcomes.

Collectively, these findings position the gut microbiota as a central mediator in alcohol-related pathophysiology, spanning hepatic, gastrointestinal, and neuropsychiatric domains. Future research should focus on precision microbiome interventions and integrative biomarker development to personalize treatment strategies for AUD and related disorders.

## Figures and Tables

**Table 1:** Selected studies on humans.

Study	Population	Age	Gender	Diagnosis	Treatment	Duration	Primary Outcomes	Microbiota Changes
Du et al. (2024) [33]	32 AUD males and 35 healthy controls (HC)	47.16 ± 9.89 (AUD participants) 48.00 ± 11.31 (controls)	Males	Severe alcohol-associated hepatitis, biopsy-confirmed	High-dose probiotic infusion, FMT, corticosteroids	23.50 (20.00, 30.00) drinking days in the past month (days)	90-day survival	Gut dysbiosis in AUD patients, and some specific microbiota, were considered to be related to alcohol intake and cognitive function. Compared with HCs, *Megamonas*, *Escherichia*, *Coprobacillus*, *Clostridium*, *Gemella*, and *Rothia* had increased in AUD patients.
Dedon et al. (2025) [34]	32 AUD patients from UConn Health in zonisamide vs. placebo RCT, 19 HC	Mean, 51 years (23–70)	47% female, 53% male	DSM-5 AUD	Zonisamide vs. placebo + behavioral therapy	16 weeks	Drinking reduction; microbiome/metabolome baseline predictors	High fecal GABA and low 3-hydroxykynurenine predicted reduction in drinking. Gut markers may forecast treatment success. ↑ *Veillonella* (worse severity), ↓ *Akkermansia*; antibiotics ↓ *Bacteroides*; steroids ↑ *Veillonella*.
Zhang et al. (2023) [35]	120 male AUD patients in Henan, China; 120 healthy controls in phase I	18–65 years	100% male	DSM-5 moderate to severe AUD	Probiotics vs. ACT vs. placebo	24 weeks (12 weeks treatment + follow-up)	AUD symptoms, craving, depression, inflammation, ERP	Protocol paper—outcomes pending. Expected comparison of psychological and microbial effects of ACT vs. probiotics. Outcomes pending but targeting ↑ *Lactobacillus*, *Bifidobacterium*; expected ↑ *Ruminococcaceae.*
Bajaj et al. (2021) [36]	20 cirrhotics with ≥2 HE episodes; randomized to SOC or SOC + FMT	65 ± 6.4 years	Males	Recurrent hepatic encephalopathy on lactulose ± rifaximin	Oral FMT capsules vs. SOC	5 months	HE recurrence, cognition, microbiota/inflammation	FMT reduced HE episodes, improved cognition, decreased IL-6 and LBP, and increased beneficial gut taxa. ↑ *Lactobacillaceae*, *Bifidobacteriaceae*; ↓ *Proteobacteria* (e.g., *Enterobacteriaceae*).
Amadieu et al. (2021) [37]	21 subjects in the placebo group and 22 in the inulin group	48.8 ± 8.8 years (placebo) group, 48.3 ± 9.8 years (inulin group)	66.2% male	DSM-IV alcohol dependence, no liver cirrhosis	Patients were supplemented with inulin (prebiotic DF) or maltodextrin (placebo)	17 days	Craving, gut permeability, cytokines, microbiota richness	Fecal metabolomics revealed 14 metabolites significantly modified by inulin versus placebo treatment (increased N8-acetylspermidine and decreased indole-3-butyric acid, 5-amino valeric acid betaine and bile acids). Fecal *Lachnoclostridium* correlated with 6 of the identified fecal metabolites, whereas plasma lipidic moieties positively correlated with fecal *Ruminococcus torques* and *Flavonifractor*.
Philips et al. (2022) [38]	50 patients with a clinical diagnosis of biopsy-proven severe	43.8 ± 9.4 years in the corticosteroid group; 48.4 ± 11.7 in the high-dose probiotic infusion (HDPI)	Male	Biopsy-proven severe alcohol-associated hepatitis	Oral prednisolone, 40 mg, once daily for 28 days	180 days	Survival at 90 and 180 days; inflammation, microbiota	Those receiving HDPI demonstrated an increased relative abundance of *Bilophila*, *Roseburia*, *Clostridium*, *Finegoldia*, *Butyricoccus*, and *Weissella* at the end of 1 month compared to baseline. *Yueomyces* was the most abundant at baseline, whereas *Myrothecium* and *Magnoliophyta* increased 1 month after high probiotic infusion.
Muthiah et al. (2021) [39]	20 healthy controls, 12 heavy-drinking controls, 11 MAH, 16 SAH, and 49 subjects with available stool (20 heavy-drinking controls, 8 MAH, 21 SAH)	46.39 ± 13.63	60% males, 40% females	Alcohol-related hepatitis or cirrhosis, MELD ≥ 15	Fresh donor FMT via nasojejunal infusion vs. SOC	6 months	Survival, infection, microbiota	*Citrobacter*, *Enterobacter*, *Puralibacter*, *Actinomycetaceae*, *Bifidobacteriaceae*, *Camobacteriaceae*, *Prevotellaceae*, *Pseudomonaceae*, *Propionibacteriaceae*, and *Veillonellaceae* positively correlated with several fecal bile acids in alcohol-associated hepatitis.
Zhang et al. (2023) [40]	60 long-term alcohol drinkers (30 placebo, 30 BC99 group) (18–65 years old, alcohol consumption ≥20 g/day, lasting for more than one year)	42.87 ± 11.15 (BC99 group), 43.60 ± 11.31 (placebo group)	99% males	DSM-5 AUD, stratified by BMI	Two groups were administered BC99 (3 g/day, 1 × 1010 CFU) or placebo (3 g/day)	30 days and 60 days	Gut microbiota recovery by BMI	BC99 regulated the imbalance of intestinal flora, increased the beneficial bacteria abundance (*Prevotella*, *Faecalibacterium*, and *Roseburia*) and reduced the conditionally pathogenic bacteria abundance (*Escherichia-Shigella* and *Klebsiella*).
Lang et al. (2020) [41]	73 ALD patients: 23 ARC, 19 AH, 24 compensated ALD + 18 controls	Median, 49.2 years (31.3–74.8)	67.1% males	Alcohol-related cirrhosis, hepatitis, or compensated ALD	None (observational)	Single time point	Microbiota, neutrophil function, inflammation	Decreased relative abundances of *Akkermansia* while the relative abundance of *Veillonella* was increased; reduction in *Bacteroides* abundance, increase in *Veillonella* abundance.
Haas et al. (2022) [42]	42 participants	Mean, 60 years	100% male	Stable coronary artery disease	Red wine (250 mL/day, 3 weeks) vs. abstinence	2 weeks wash-out period	Plasma TMAO, gut microbiota, metabolome	Red wine did not affect TMAO but shifted microbiota and metabolome. ↑ *Parasutterella, Bacteroides*, *Prevotella*; no change in TMAO-producing bacteria.
Han et al. (2015) [43]	117 hospitalized AH patients: 60 probiotic, 57 placebo	Mean, 52.7 ± 11.3 years	64% male	Mild alcoholic hepatitis, non-cirrhotic, recent drinking	*L. subtilis* + *S. faecium* vs. placebo + silymarin	7 days (inpatient)	LPS, TNF-α, IL-1β, liver enzymes	Probiotics reduced TNF-α, LPS, and stool *E. coli* CFUs. Greatest benefit in cirrhotics. Additive to abstinence effects. ↓ *E. coli* CFU; probiotics modulated gut flora positively, especially in cirrhotics.

↑ indicates an increase in bacterial abundance or clinical parameter; ↓ indicates a decrease.

**Table 2:** Selected studies on animals.

Study	Animal	Strain	Sex	Sample Size	Intervention	Dose	Exposure Duration	Key Microbiota Findings	Other Observations
Daaz-Ubilla et al. (2025) [44]	Rat	Wistar	Males and female	10 per group	bEVs from ethanol-naïve and ethanol-exposed UChB rats injected intraperitoneally (3 days)	11.4 ± 1.2 g ethanol/kg/day for 120 days	120 days exposure in donors, 3-day bEV exposure in recipients, followed by 4-day test	Exposure to blood extracellular vesicles (bEVs) derived from ethanol-exposed UChB rats resulted in significant changes in the gut–brain axis of naïve Wistar rats. Although the bEVs did not induce systemic inflammation or changes in microglial activation, they triggered microbiota-brain interactions that increased ethanol-seeking behavior.	Behavioral tests (two-bottle choice) showed increased voluntary ethanol consumption in bEV-exposed Wistar rats. This effect was abolished when the vagus nerve was surgically cut, highlighting vagal involvement. No significant IL-6, TNF-α, or CD11b elevation in liver or brain tissues.
Xu et al. (2018) [45]	Mouse	C57BL	Male	Varied: n = 6 for preliminary, n = 31 alcohol group, n = 16 control (main test)	Oral ethanol in drinking water with gradient concentrations	The concentration of alcohol was increased from 2%, 4%, to 6% every 3 days and reached 8%	21 days	Microbiota profiling using 16S rRNA sequencing showed that ethanol consumption significantly ↑ *Actinobacteria* and *Cyanobacteria phyla*. At the genus level, ↑ *Adlercreutzia*, *Allobaculum*, and *Turicibacter* were noted and ↓ *Helicobacter*.	Ethanol-treated mice displayed reduced locomotor activity, higher immobility in tail suspension and forced swim tests, and decreased open-arm exploration in the elevated plus maze, suggesting anxiety- and depression-like phenotypes. These behaviors were associated with downregulation of BDNF and Gabra gene expression in both hippocampus and prefrontal cortex.
Xiao et al. (2018) [46]	Mouse	C57BL	Male	n = 12 per group (water group and alcohol group)	Gavage alcohol feeding: week-wise 5% to 35% ethanol; then, withdrawal	Water for mice in water group; 5%, 10%, 20%, 35% alcoholic solution force-fed into the mice’s stomach for the alcohol group	4 weeks	Increased *Erysipelotrichia*, *Erysipelotrichaceae*, and *Erysipelotrichales*, whereas *Lactobacillaceae*, *Lactobacillus*, *Lactobacillale*, *Bacilli*, *Bacteroides*, *Parabacteroides*, and *Alloprevotella* were significantly reduced.	Alcohol withdrawal in donor mice increased immobility in forced swim and tail suspension tests, decreased sucrose preference, and increased anxiety scores. FMT alone was sufficient to transfer these behaviors.
Wang et al. (2018) [47]	Mouse	BALB/c	Female	n = 10 per group, 3 groups	Active vs. forced alcohol drinking (3% → 20% over 7 weeks; then, withdrawal)	3%, 6%, 10%, 20% alcohol progressively	8 weeks (7 weeks alcohol + 1 week withdrawal)	During active alcohol exposure, there was a marked ↑ in *Firmicutes* and *Clostridiales*, as well as specific genera like *Lachnospiraceae*, *Alistipes*, and *Odoribacter*. These changes persisted after withdrawal, indicating long-term dysbiosis. Concurrently, there was increased serotonin concentration in the gut.	Histological examination revealed hepatocellular degeneration and colonic epithelial damage in alcohol-exposed mice. Behavioral assessments post-withdrawal showed significant anxiety and depression. These included decreased center time in open-field tests and increased immobility.
Li et al. (2022) [48]	Rat	Wistar	Male	n = 20 per group, 3 groups	Gavage ethanol (8 → 12 mL/kg/day) for 12 weeks; co-exposure with dietary iron	56% ethanol, 8–12 mL/kg/day	12 weeks	Rats exposed to both alcohol and high dietary iron experienced significant intestinal dysbiosis characterized by ↓ *Lactobacillus* and ↑ *Bacteroides* and *E. coli*. Supplementation with *Lactobacillus casei* reversed these alterations, restoring microbial balance towards homeostasis.	Combined alcohol and iron exposure led to elevated serum ferritin, hepcidin, and increased protein expression of intestinal DMT1 and FPN1—indicators of iron overload. *L. casei* supplementation significantly reduced these markers.
Yang et al. (2024) [49]	Mouse	C57BL/6J	Male	20 pubertal (P27–P44), 20 adult (P60–P78)	20% ethanol in sterile water	20%	2 weeks (chronic exposure); fecal samples collected at 0 h, 24 h, 1 week, 2 weeks	In pubertal mice: mild gut dysbiosis; ↓ in *Lactobacillus intestinalis* and *Limosilactobacillus reuteri*; ↑ in *Bifidobacterium*, *Butyricimonas*, and *Alistipes shahii*; ↓ in *Turicimonas muris* and *L. taiwanensis.* In adult mice: more severe dysbiosis with ↑ *Alistipes*, *Bacteroides*; ↓ *Lactobacillus*, *Mucispirillum schaedleri*.	Pubertal mice showed less liver and intestinal injury, increased ALDH activity, decreased ADH. Adult mice had increased mucin-degrading enzymes, liver enzyme imbalance, higher oxidative stress enzymes, and more histological damage to small intestine.
Yi et al. (2023) [50]	Mouse	C57BL/6J	Male	56 (7 groups, 8 mice each)	5% ethanol in drinking water for 10 days; then, 31.5% ethanol via gavage on day 102	5% (daily ethanol); 31.5% (gavage ethanol)	13 weeks + 10 days ethanol feeding + 1 ethanol gavage	Citrus honey (CH) reversed ethanol-induced gut dysbiosis: ↑ *Bacteroidota*; ↓ *Firmicutes*, *Proteobacteria*, *Verrucomicrobiota*, and *Turicibacter*. Improved SCFA levels (acetic, propionic, butyric, and valeric acids).	CH decreased ALT and AST levels, protected against alcohol-induced liver histopathology (reduced steatosis and inflammation). Dose-dependent effects seen with LH and HH. CH effects were superior to fructose syrup.
Xia et al. (2021) [51]	Mouse	ICR	Male	8–10 mice per group (control group, model group, low- and high- dose ZAVE groups)	Oral gavage (daily)	Escalating: 2 g/kg (week 1), 4 g/kg (week 2), 6 g/kg (days 15–30)	30 days	ZAVE ↑ Akkermansia, *Lachnospiraceae*, and *Bacteroidetes*; ↓ *Firmicutes*, *Proteobacteria*, *Bilophila*, and *Butyricimonas*. Reversed ethanol-induced dysbiosis and improved F/B ratio.	Improved gut barrier, increased IL-10, TGF-β, IgA, IL-22, Reg3b/g. Reduced ROS, LPS, TNF-α, IL-6, IL-1β, liver enzymes (ALT/AST), and histological damage.
Yang Fan et al. (2018) [52]	Rat	Wistar	Male	40 total (n = 6 per group selected for sequencing)	In drinking water	Gradually from 1% to 6% (then maintained at 6%)	30 days + withdrawal (up to 14 days)	No significant diversity/richness changes; colon: ↑ *Bacteroidetes*, *Ruminococcaceae*, *Parabacteroides*, *Butyricimonas*, ↓ *Lactobacillus*, *Gauvreauii*; Jejunum mostly unaffected.	Alcohol dependence significantly alters colonic microbiota; microbiota partially restored after withdrawal; KEGG functions ↑ in amino acid metabolism, peroxisome, polyketide sugar biosynthesis; behavioral withdrawal signs observed.
Hendrikx et al. (2020) [53]	Chronic–binge ethanol feeding (NIAAA model)	C57BL/6 mice and Reg3g^−^/^−^ mice	Males and females	WT: n = 51; KO: n = 46 (cumulative across groups)	Lieber–DeCarli ethanol diet, followed by gavage with 5 g/kg ethanol	~36% of total calories from ethanol (starting day 6)	15 days total (10 days ethanol, final gavage on day 16)	Ethanol feeding reduced levels of indole-3-acetic acid (IAA), an AHR ligand; impaired IL22 and REG3G expression; increased bacterial translocation to liver. Engineered *L. reuteri*/IL22 restored IL22 and REG3G, reduced dysbiosis-associated liver damage.	Antibiotics restored IL22 expression and reduced damage. IAA supplementation increased IL22 and REG3G, prevented bacterial translocation. Engineered *L. reuteri*/IL22 strain showed therapeutic potential. No protective effect observed in Reg3g^−^/^−^ mice.
Chen et al. (2015) [54]	Mouse	C57BL/6	Female	GF: 7–16, Conv: 10–15	Single oral gavage	30% ethanol (vol/vol)	Single binge (sacrificed after 9 h)	Germ-free mice showed exacerbated liver injury, inflammation, and steatosis despite lower blood ethanol levels due to increased ethanol metabolism. No changes in microbiota after single binge in conventional mice.	Higher expression of Adh1, Aldh2, CYP2E1, Srebp-1, and increased hepatic triglycerides in GF mice. GF mice had heightened baseline hepatic inflammation and upregulated proinflammatory cytokines.
Yang et al. (2021) [55]	Mouse	C57BL/6	Male	6 mice per group	Chronic ethanol feeding plus binge (NIAAA model)	5% (*v*/*v*) ethanol Lieber–DeCarli diet + 5 g/kg binge	10 days Lieber–DeCarli + 1 binge (day 11)	Ethanol-fed mice showed decreased abundance of beneficial genera such as *Lactobacillus* and increased abundance of potentially pathogenic taxa like *Escherichia-Shigella* and *Enterococcus*. Overall microbial diversity was reduced. *Probiotic Clostridium butyricum* reversed dysbiosis.	Ethanol feeding elevated intestinal permeability and inflammation (TNF-α, IL-1β), while probiotic intervention restored tight junction proteins (ZO-1, occludin), reduced serum ALT/AST, and alleviated liver steatosis and oxidative stress. *Clostridium butyricum* modulated TLR4/NF-κB signaling.
Xue et al. (2017) [56]	Mouse	C57BL/6J	Male	45 mice (15 per group—control, ethanol, aplysin, and ethanol)	Control or ethanol-containing liquid diet with varying protein sources; final dose involved binge ethanol gavage	8 mL ethanol/kg for 2 weeks; then, 12 mL/kg for 6 weeks	Collection of liver and cecum samples for analysis	SPI and hydrolyzed SPI diets enriched beneficial gut microbes (*Allobaculum*, *Bifidobacterium*, *Lactobacillus*, *Akkermansia*) and reduced ethanol-associated increases in *Helicobacter, Anaeroplasma*, and *Proteobacteria*. Metagenomic prediction indicated enhanced bile acid metabolism and SCFA biosynthesis.	SPI diets improved liver histology: ↓ steatosis, ↓ inflammation, ↓ ALT/AST vs. casein group. Tight junction proteins were upregulated. SPI diets also modulated bile acid profiles and nuclear receptor pathways with downstream effects on lipid metabolism and hepatic inflammation.
Mittal et al. (2025) [57]	Mouse	C57BL/6N	Male	n = 8 per group for microbiome and biochemical studies; n = 3 per group for liver proteomic analysis due to cost constraints	Lieber–DeCarli ethanol diet to induce ALD, combined with intraperitoneal thioacetamide injections (150 mg/kg body weight, twice weekly) to enhance hepatic injury	20–22% of total caloric intake derived from ethanol, consistent with standard ALD induction protocols	Initial 1-week acclimatization on liquid diet, followed by 8 weeks of Lieber–DeCarli + ethanol + thioacetamide. Post-alcohol abstinence phase involved 7 days of dietary intervention with standard, egg-based, or plant-based diet	Veg diet group showed significant enrichment of beneficial microbial taxa: *Lachnospiraceae*, *Prevotellaceae*, *Kurthia*, *Christensenellaceae*, *Akkermansia*, and *Butyricicoccus*. It also decreased pathogenic bacteria: *Roseburia, Klebsiella*, *Staphylococcus*, and *Pseudomonas* vs. egg diet group. Functional shifts included ↑NAD salvage pathway, glycolysis, TCA cycle, and urea cycle.	Vegetable protein diet significantly reduced hepatic steatosis compared to the egg diet. ALT and AST serum levels were reduced vs. egg diet. Proteomics revealed upregulation of recovery-related metabolic pathways, including fatty acid beta-oxidation, pyruvate, methionine, and cysteine metabolism. Co-expression analysis (WGCNA) showed strong correlation between veg diet and upregulated energy metabolism and antioxidant pathways.
Thoen et al. (2022) [58]	Wistar rats	Adults	Males	24 (8 per group: control, ALC4, ALC8)	10% ethanol + sunflower seed diet + binge (5 g/kg, gavage)	10%	4 weeks (ALC4), 8 weeks (ALC8)	↑ *Bacteroidetes*, ↑ *Proteobacteria*, ↓ *Firmicutes*; correlated with liver markers (TG, ALT, AST, albumin, steatosis).	ALC4: Grade 2 micro, Grade 1 macro steatosis; ALC8: Grade 3 micro, Grade 1 macro steatosis; no fibrosis; ↑ AST, ALT, glucose; ↓ albumin, HDL-C; significant mortality in ALC8.
Jiang et al. (2019) [59]	Mouse	C57BL/6J	males	21 mice (3 groups of 7); plus 18 FMT recipients (3 × 6 mice)	Oral ethanol via drinking water (4 days/week)	15% (*v*/*v*)	10 weeks	↑ *Akkermansia*, *Clostridium* in alcohol group, ↓ *Prevotella*, *Barnesiella*, *Alloprevotella*, *Alistipes*. Strong correlation between inflammatory cytokines (IL-1β, IL-6, TNF-α, IL-10, TGF-β) and microbial genera.	Alcohol caused depressive-like behavior; nicotinamide riboside (NR) improved behavior and anxiety. Alcohol increased microglial activation (CD68↑); NR reduced it.
Zhang et al. 2019) [60]	Rhesus macaques	Macaca mulatta	Males	12 total; alcohol drinking or control groups of adolescent alcohol (n = 6), adolescent control (n = 6), adult alcohol (n = 4), and adult control (n = 5)	Custom-designed operant drinking panel attached to one side of the cage	4% *w*/*v* ethanol solution (0.5 g/kg; then, 1 g/kg; then, 1.5 g/kg;)	3 months	Ethanol-exposed animals had ↑ *Bacteroidetes*, *Firmicutes*, *Tenericutes, Actinobacteria*, *Proteobacteria*, and *Spirochaetes*.	The effects of ethanol-exposed group were partially or wholly ameliorated following a relatively short 5-day period of abstinence, suggesting that the specific effects observed here are the direct effects of alcohol.
Cunningham et al. (2023) [61]	Mouse	C57BL/6	Males and females	9 breeding pairs in each group (AUDIT score > 8 and AUDIT score < 8, respectively)	Mice were colonized with human fecal microbiota from individuals with high and low AUDIT scores and bred to produce human alcohol-associated microbiota or human control-microbiota	Human positive fecal samples from subjects with AUDIT score of ≥8 for men and ≥5 for women	Last alcohol-containing beverage consumed within the 7 days prior to enrollment	↑ *Klebsiella pneumoniae*, ↑ *Streptococcus pneumoniae*.	Offspring colonized with fecal microbiota from high-AUDIT adults exhibited higher mortality, pulmonary bacterial burden, and post-infection lung damage to *Klebsiella pneumoniae* and *Streptococcus pneumoniae* pneumonia.
Wang et al. (2023) [62]	Rat model	Antibiotics-treated conventional rats	Male	n = 8 per group; groups included control, ethanol-fed, and ethanol- + LGG-treated mice	Role of the gut microbiome on the behaviors of rats by fecal microbiota transferred orally throughout ethanol treatment	489.42 ± 29.91 alcohol intake/day (15.82 ± 9.04 years) for humans from which fecal microbiota was collected	FMT daily for 21 days, behavioral testing for the next 6 days, alcohol preference test for the next 5 days	↑ *Lactobacillus*, ↑ *Akkermansia*, ↑ *Rikenellaceae*; ↓ *Enterobacteriaceae,* and ↓ *Bacteroides.*	Alcohol dependence in rats, including increased anxiety- and depression-like behaviors, reduced exploratory and recognition memory, and higher alcohol preference.

↑ indicates an increase in bacterial abundance or clinical parameter; ↓ indicates a decrease; AUDIT—Alcohol Use Disorders Identification Test.

**Table 3:** Percentage breakdown by diagnosis; proportion of total participants across the 11 human studies.

Diagnosis	Participants	Percentage (%)
Alcohol use disorder	289	41.17%
Alcoholic hepatitis	203	28.91%
Alcohol-related cirrhosis	98	13.96%
Hepatic encephalopathy (HE)	20	2.85%
Coronary artery disease (CAD)	42	5.98%
Other/mixed (AH + ARC or ALD)	50	7.13%

**Table 4:** Effect size estimates for human studies evaluating interventions or outcomes related to alcohol use and microbiota.

Study	Outcome	Cohen’s d	Hedges’ g
Du et al. [33]	MoCA (AUD vs. HC)	−0.98	−0.96
	MMSE (AUD vs. HC)	−1.25	−1.22
Dedon et al. [34]	Percent drinking reduction (placebo vs. zonisamide)	0.39	0.38
Zhang et al. [35]	Protocol only, no outcome data available	No data available	No data available
Bajaj et al. [36]	ACQ-SF score at day 15 (FMT vs. placebo)	0.29	0.28
Amadieu et al. [37]	Metabolomics outcomes only	No data available	No data available
Philips et al. [38]	90-day survival (FMT vs. HDPI)	0.69 (large)	
Muthiah et al. [39]	Metabolomics/microbiome data only	No data available	No data available
Zhang et al. [40]	AST at 60 days (BC99 vs. placebo)	0.66	0.65
	γ-GT at 60 days (BC99 vs. placebo)	0.87	0.86
Lang et al. [41]	Observational microbiome outcomes	No data available	No data available
Haas et al. [42]	Crossover metabolomics data only	No data available	No data available
Han et al. [43]	Change in serum LPS (probiotics vs. placebo, day 7)	0.18	0.18

**Table 5:** Summary of human studies based on type of intervention and primary microbiota findings.

Treatment Type	Number of Studies	Microbiota Outcome
FMT	4	↑ *Diversity*, ↓ *Proteobacteria*
Probiotics	2	↑ *Lactobacillus*, ↓ *E. coli*
Abstinence	2	Partial recovery in SCFA-producers
Pharmacologic (Zonisamide [34], Pentoxifylline [38])	2	Predictive microbial/metabolite markers
Observational	1	↓ *Ruminococcaceae*, ↑ craving and zonulin

↑ indicates an increase in bacterial abundance or clinical parameter; ↓ indicates a decrease.

**Table 6:** Microbial changes by study (humans).

Bacteria	Change	Study
*Megamonas*	Increase	Du et al. [33]
*Escherichia*	Increase	Du et al. [33]
*Coprobacillus*	Increase	Du et al. [33]
*Lactobacillus*	Decrease	Du et al. [33]; Philips et al. [38]; Bajaj et al. [36]
*Fungal species*	Increase	Du et al. [33]
*Veillonella*	Increase	Dedon et al. [34]
*Akkermansia*	Decrease	Dedon et al. [34]
*Bacteroides*	Decrease	Dedon et al. [34]; Haas et al. [42]
*Lactobacillaceae*	Increase	Bajaj et al. [36]
*Bifidobacteriaceae*	Increase	Bajaj et al. [36]
*Proteobacteria*	Decrease	Bajaj et al. [36]; Philips et al. [38]
*Ruminococcaceae*	Increase	Amadieu et al. [37]; Muthiah et al. [39]; Zhang et al. [35]
*Lachnospiraceae*	Decrease	Amadieu et al. [37]; Lang et al. [41]
*Faecalibacterium*	Increase	Philips et al. [38]; Zhang et al. [35]
*Enterobacteriaceae*	Decrease	Muthiah et al. [39]
*Enterococcus*	Increase	Lang et al. [41]
*Streptococcus*	Increase	Lang et al. [41]
*Parasutterella*	Increase	Haas et al. [42]
*Prevotella*	Increase	Haas et al. [42]
*E. coli*	Decrease	Han et al. [43]

**Table 7:** Microbial changes by study (animals).

Bacteria	Change	Study
*Actinobacteria*	Increase	Xu et al. [45]
*Cyanobacteria*	Increase	Xu et al. [45]
*Adlercreutzia*	Increase	Xu et al. [45]
*Allobaculum*	Increase	Xu et al. [45]; Xue et al. [56]
*Turicibacter*	Increase	Xu et al. [45]
*Helicobacter*	Decrease	Xu et al. [45]; Xue et al. [56]
*Firmicutes*	Increase	Wang et al. [47]
*Clostridiales*	Increase	Wang et al. [47];
*Lachnospiraceae*	Increase	Wang et al. [47]; Mittal et al. [57]
*Alistipes*	Increase	Wang et al. [47]; Jiang et al. [59]
*Odoribacter*	Increase	Wang et al. [47]
*Lactobacillus*	Decrease	Li et al. [48]; Yang et al. [49]
*Bacteroides*	Increase	Li et al. [48]; Wang et al. [62]
*E. coli*	Increase	Li et al. [48]
*Akkermansia*	Increase	Mittal et al. [57]; Jiang et al. [59]
*Bifidobacterium*	Increase	Xue et al. [56];
*Anaeroplasma*	Decrease	Xue et al. [56]
*Proteobacteria*	Decrease	Xue et al. [56];
*Roseburia*	Decrease	Mittal et al. [57]
*Klebsiella*	Decrease	Mittal et al. [57]; Cunningham et al. [61]
*Staphylococcus*	Decrease	Mittal et al. [57]
*Pseudomonas*	Decrease	Mittal et al. [57]
*Escherichia-Shigella*	Increase	Yang et al. [49]; Zhang et al. [60]
*Enterococcus*	Increase	Yang et al. [49]; Zhang et al. [60]
*Prevotella*	Decrease	Jiang et al. [59]
*Barnesiella*	Decrease	Jiang et al. [59]
*Alloprevotella*	Decrease	Jiang et al. [59]
*Ruminococcaceae*	Decrease	Zhang et al. [60]
*Lachnospiraceae*	Decrease	Zhang et al. [60]
*Enterobacteriaceae*	Decrease	Wang et al. [62]
*Rikenellaceae*	Increase	Wang et al. [62]
*Streptococcus*	Increase	Cunningham et al. [61]

**Table 8:** Discrepancies in microbial findings.

Humans		Animals	
*Akkermansia*	Decrease [34]	*Akkermansia*	Increase [57,59,61]
*Bacteroides*	Decrease [34,42]	*Bacteroides*	Increase [48,62]
*E. coli*	Decrease [43]	*E. coli*	Increase [48]
*Lachnospiraceae*	Decrease [37,41]	*Lachnospiraceae*	Increase [60]
*Prevotella*	Increase [42]	*Prevotella*	Decrease [59]
*Roseburia*	Increase [33]	*Roseburia*	Decrease [57]

**Table 9:** Effect size estimates for animal model studies assessing behavioral, biochemical, and microbiota-related outcomes in alcohol exposure models.

Study	Outcome	Cohen’s d	Hedges’ g
Daaz-Ubilla et al. [44]	Ethanol intake (g/kg/day)	5.31	4.79
	Ethanol intake (g/kg/day)	5.01	4.52
Xu et al. [45]	OFT—time in inner zone	0.69	0.68
	EPM—time in open arms	0.93	0.90
Xiao et al. [46]	FST—immobility time	1.84	1.71
	TST—immobility time	2.04	1.90
Wang et al. [47]	Light–dark test—time in dark	1.19	1.10
	Open field—distance traveled	5.10	4.69
	Open field—time in center	3.85	3.54
Li et al. [48]	Serum ferritin (ng/mL)	1.10	1.01
	Serum hepcidin (ng/mL)	1.47	1.35
Yang et al. [49]	ALT (U/L)—pubertal mice	1.57	1.46
	ALT (U/L)—adult mice	2.21	2.06
	AST (U/L)—pubertal mice	1.33	1.24
	AST (U/L)—adult mice	1.50	1.39
Yi et al. [50]	ALT (U/L)	1.28	1.19
	AST (U/L)	1.40	1.30
Xia et al. [51]	ALT (U/L)	4.04	3.76
	AST (U/L)	3.67	3.41
Yang Fan et al. [52]	Withdrawal severity score	2.34	2.18
Hendrikx et al. [53]	Plasma ALT (U/L)	1.07	1.00
Chen et al. [54]	Plasma ALT (U/L)	1.86	1.73
Yang et al. [55]	D-lactate (μmol/L)	1.77	1.65
	DAO (ng/mL)	1.70	1.58
	LPS (EU/L)	3.43	3.19
Xue et al. [56]	ALT (U/L)	0.67	0.62
	AST (U/L)	0.76	0.71
Mittal et al. [57]	ALT (U/L)	2.45	2.28
	AST (U/L)	1.49	1.38
Thoen et al. [58]	ALT (U/L)	5.38	5.00
	AST (U/L)	13.47	12.53
Jiang et al. [59]	SPT (%)	3.31	3.08
	FST (s)	2.30	2.14
Zhang et al. [60]	Chao1 index	2.75	2.56
	Shannon index	1.94	1.80
Cunningham et al. [61]	Bacterial burden (log CFU/lung)—*K. pneumoniae*	1.23	1.14
	Bacterial burden (log CFU/lung)—*S. pneumoniae*	1.64	1.53
Wang et al. [62]	OFT—time in center	2.21	2.05
	EPM—time in open arms	1.81	1.68
	FST—immobility time	1.80	1.67

## Data Availability

No new data were created or analyzed in this study. Data sharing is not applicable to this article.
